# Characterization of two *Lactococcus lactis* zinc membrane proteins, Llmg_0524 and Llmg_0526, and role of Llmg_0524 in cell wall integrity

**DOI:** 10.1186/s12866-015-0587-1

**Published:** 2015-10-30

**Authors:** Célia Roussel, Bénédicte Cesselin, Rémy Cachon, Philippe Gaudu

**Affiliations:** INRA, UMR1319 Micalis, F-78350 Jouy-en-Josas, France; AgroParisTech, UMR Micalis, F-78350 Jouy-en-Josas, France; UMR A 02.102 Unité Procédés Alimentaires et Microbiologiques, AgroSup Dijon-Université de Bourgogne, 1-esplanade Erasme, F-21000 Dijon, France; Institut Micalis UMR1319 et AgroParisTech, Domaine de Vilvert, 78352 Jouy-en-Josas, Cedex, France

**Keywords:** Cysteine, Zinc, Membrane proteins, Growth, Cumene hydroperoxide, Lysozyme

## Abstract

**Background:**

Due to its extraordinary chemical properties, the cysteine amino acid residue is often involved in protein folding, electron driving, sensing stress, and binding metals such as iron or zinc. *Lactococcus lactis*, a Gram-positive bacterium, houses around one hundred cysteine-rich proteins (with the CX_2_C motif) in the cytoplasm, but only a few in the membrane.

**Results:**

In order to understand the role played by this motif we focused our work on two membrane proteins of unknown function: Llmg_0524 and Llmg_0526. Each of these proteins has two CX_2_C motifs separated by ten amino-acid residues (CX_2_CX_10_CX_2_C). Together with a short intervening gene (*llmg_0525*), the genes of these two proteins form an operon, which is induced only during the early log growth phase. In both proteins, we found that the CX_2_CX_10_CX_2_C motif chelated a zinc ion via its cysteine residues, but the sphere of coordination was remarkably different in each case. In the case of Llmg_0524, two of the four cysteines were ligands of a zinc ion whereas in Llmg_0526, all four residues were involved in binding zinc. In both proteins, the cysteine-zinc complex was very stable at 37 °C or in the presence of oxidative agents, suggesting a probable role in protein stability. We found that the complete deletion of *llmg_0524* increased the sensitivity of the mutant to cumene hydroperoxide whereas the deletion of the cysteine motif in Llmg_0524 resulted in a growth defect. The latter mutant was much more resistant to lysozyme than other strains.

**Conclusions:**

Our data suggest that the CX_2_CX_10_CX_2_C motif is used to chelate a zinc ion but we cannot predict the number of cysteine residue involved as ligand of metal. Although no other motif is present in sequence to identify roles played by these proteins, our results indicate that Llmg_0524 contributes to the cell wall integrity.

**Electronic supplementary material:**

The online version of this article (doi:10.1186/s12866-015-0587-1) contains supplementary material, which is available to authorized users.

## Background

Cysteine is an extraordinary amino acid residue because of the chemical reactivity of its thiol group (SH). In contact with oxidative compounds, the thiol group can oxidize itself into sulfenic acids (SOH) or form a disulfide bond (R-S-S-R’) with another cysteine residue, either from the same or from a different protein. These oxidized states can revert back to the reduced form (R-SH) via the action of an electron donor [1, 2]. In contrast, autooxidation into sulfinic (SO_2_H) and sulfonic acid (SO_3_H) leads to irreversible states of cysteine. Besides cysteine also enables the sequestration of redox metals alone or in complex structure like iron-sulfur cluster that can modulate activity of the protein [3]. This amino acid can also bind non-redox metals such as zinc ion, for example in the zinc finger complex, thus enabling the correct folding of proteins. It is worth noting this latter complex is mainly found in eukaryotic cells [4]. Due to the redox properties of cysteine, the oxidation of this amino acid can activate transcriptional factors directly, as has been described in *E. coli* (OxyR, [5]) and *Bacillus subtilis* (OhrR, [6]), as well as indirectly, via the oxidation of a [Fe/S] cluster as described in SoxR and FNR in *Escherichia coli*. SoxR detects superoxide anions via oxidation of its [2S/2Fe] cluster, while FNR detects oxygen via oxidation-destruction of its [4S/4Fe] [3]. In addition, cysteine residues have also been associated with enzymatic activities, such as those involving glyceraldehyde dehydrogenase (Gapdh, a metabolic enzyme, [7]), heat shock chaperone proteins (Hsp33 [8], DnaJ [9]), and thioredoxins and glutaredoxins (electron carriers [2]). Thus, because of their involvement in multiple key aspects of cellular metabolism, cysteine residues in proteins should be widely distributed across all organisms, from viruses to complex forms like the eukaryotic cells.

Unexpectedly, recent studies on extracytoplasmic proteins whose activation is mediated by intramolecular disulfide bonds revealed that proteins in Gram-positive bacteria are proportionately poorer in cysteine residues than proteins found in other bacteria, a phenomenon termed “cysteine exclusion” [10, 11]. One proposed explanation was that, unlike Gram-negative bacteria, Gram-positive bacteria have neither a periplasmic space nor outer membrane. Thus, exported cysteine-rich proteins would be subject to oxidation, which, in the absence of repair machinery such as the DsB system in *E. coli* [1], would lead to their inactivation. In Gram-positive bacteria, some systems have been described: BdB system in *B. subtilis* [12] and more recently in *Mycobacterium tuberculosis* [13, 14] and in *Streptococcus gordonii* [15].

Compared to *E. coli* or *B. subtilis*, in *Lactococcus lactis*, a facultative aerobic bacterium of high technological interest, such system of repair has not yet been reported although this organism is widely used to overproduce recombinant proteins like cytokine IL-12 containing disulfide bridge for its activity [16]. Unexpectedly, this organism has an even lower amount of cysteine residue in its proteins [10, 11, 17]. Thus far, investigations of the proteins of this bacterium have characterized only a few whose cysteine residues interact with an iron-sulfur cluster or catalyze reduction of substrates, and all of these are cytosolic proteins. In the activase protein (NrdG) of the anaerobic ribonucleotide reductase complex (RNR), these residues are predicted to sequestrate a [4Fe/4S] cluster [18] and only three cysteines might be engaged to complex it as reported in *E. coli* NrdG [19]. In the dihydroorotate dehydrogenase (PyrK), four cysteines sequester a [2Fe/2S] redox cluster [20]. Both RNR and PyrK are connected to DNA synthesis. In the alkylhydroperoxide reductase (AhpC/AhpF) [21] and thioredoxin reductase/thioredoxin system (TR/Trx) [7], cysteines directly catalyze the reduction of substrates. AhpC/AhpF is one of the few enzymes described so far that play a role in oxidative stress resistance. TR/Trx maintains the intracellular redox state to protect intracellular proteins (like Gapdh) against oxidation [7, 22]. Finally, Clp ATPase (ClpE) was reported to contribute to the degradation of misfolded or truncated intracellular proteins [23], and its activity could depend on a putative cysteine-containing zinc finger [24].

In this work, we aimed to understand role of cysteines in *L. lactis* proteins and role of these proteins when cells are exposed to stressful conditions. Using a bioinformatic approach, we selected cysteine-rich proteins located in the membrane as they might constitute a first signaling pathway to detect environmental stress, or a defense line, through cysteine oxidation. Among a few proteins found in *L. lactis* strain MG1363, we focused on Llmg_0524 and Llmg_0526, of which respective genes form an operon including also a small gene*, llmg_0525*. This operon was transiently induced at very early log growth phase. In the proteins, cysteine residues are organized in a CX_2_CX_10_CX_2_C motif involved in zinc coordination. Finally, through different constructions in Llmg_0524 we found that this protein had a severe impact on growth, affected resistance to cumene hydroperoxide and lysozyme suggesting this protein is linked to cell envelope integrity.

## Results

### *In silico* analysis of the cysteine-rich membrane proteins

To identify the predicted membrane proteins that contained the CX_2_C motif in *L. lactis* strain MG1363, we used a bioinformatic approach. From the genome database we found that proteins harboring the potential redox CX_2_C motif represented only 4.88 % of total proteins (122 out of 2,434 proteins) (see *in silico* analysis section of Methods). This score is the lowest when we compared it to 6.39 % found in *B. subtilis* (strain 168, 267 out of 4,175 proteins) or 10.04 % in *E. coli* (strain K-12 subsp MG1655, 416 out of 4,141 proteins). These data are in agreement with the theory of “cysteine exclusion” and is also strengthened by the capacity of *L. lactis* strain to grow in presence of large amount of reducing agents like dithiothreitol (DTT) [10, 25].

When we used the prediction program (TMHMM server) to extract only membrane proteins, this number dropped to seven against 42 found in *B. subtlilis* or 94 in *E. coli*. Three of these proteins had a predicted function, whereas the remaining four did not (Table 1). Llmg_0199 (FeoB) and Llmg_1729 (CopA) have been linked to metal (iron and copper, respectively) homeostasis in bacteria [26–28]. The former protein is particularly likely to serve an essential function, as iron is necessary in *L. lactis* not only for DNA synthesis (with RNR and PyrK) but also for heme synthesis from protoporphyrinogen IX [29]. Heme enables *L. lactis* to undergo respiration, and this growth condition (aerated medium supplemented with heme) translates into increased biomass yield and an increase in long-term survival versus fermentation [29, 30]. Llmg_2304 (ComC) contained six cysteines, including two CX_2_C motifs separated by 21 amino acid residues. This protein is similar to PilD (23 % of identity, mainly in the N-terminal extremity), a peptidase involved in pili synthesis. In *Pseudomonas* species, the cysteine residues of PilD should complex a zinc ion. However, studies revealed that the cysteine-zinc complex may be unstable *in vitro* as, in some protein preparations, a disulfide bond was formed instead [31, 32]. Llmg_0524 and Llmg_0526 each contained four cysteines, in a CX_2_CX_10_CX_2_C motif in the N-terminal extremity (N^ter^) (Additional file 1: Figure S1), while Llmg_1066 harbored a CX_2_C motif in its C-terminal extremity (C^ter^). Llmg_1102 contained four target motifs in its N^ter^ domain, which were organized into two CX_2_CX_14_CX_2_C motifs. With the exception of the cysteine motif, no other signatures were detected that predicted the function of the latter four proteins.

**Table 1:** Putative membrane proteins containing CX_2_C motif in *L. lactis* strain MG1363

Proteins	TMD	N. of cysteine	N. of CX_2_C motif
Llmg_0199, FeoB	12	5	1
Llmg_1729, CopA	8	4	1
Llmg_2304, ComC	6	6	2
Llmg_0524	2	4	2
Llmg_0526	1	4	2
Llmg_1066	2	2	1
Llmg_1102	1	8	4

TMD, transmembrane domain (TMHMM prediction program, cut off >0.4)

To understand the role of the cysteine motif in *L. lactis* proteins, we continued our investigation by focusing on two cysteine-rich proteins, Llmg_0524 and Llmg_0526, which might sense stressful environments via cysteine oxidation and their putative membrane localization. We aimed to determine: i) the genetic organization and expression of these proteins; ii) the localization and properties of the cysteine motif within the proteins; and iii) the role of these proteins.

### Temporal expression of the llmg_0524-0525-0526 operon

**Fig. 1:**
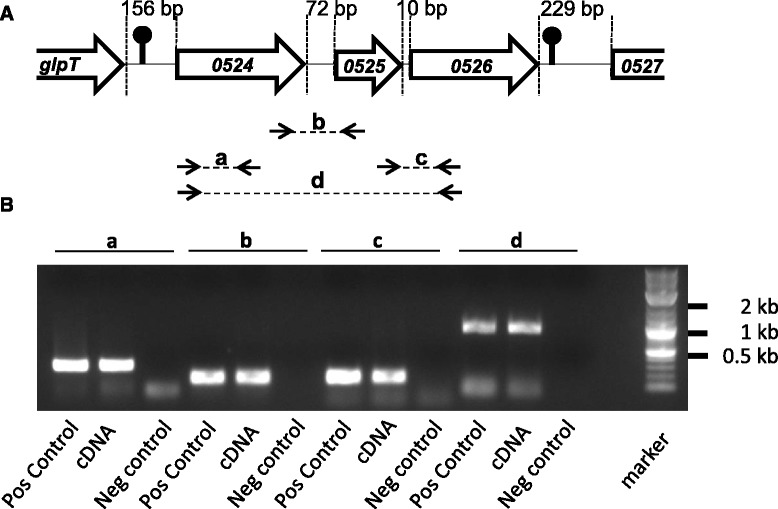
*llmg_0524*, *llmg_0525*, and *llmg_0526* form an operon. **a** Schematic representation of the locus; bp - base pair. **b** RT-PCR analysis of the locus. Total RNA was translated into cDNA, which in turn was used as the template for PCR with different primer pairs (*a*, *b*, *c*, and *d*). Pos Control, positive control with genomic DNA as a template; Neg Control, negative control with treatment of RNA preparation with a RNase

We found a stem loop upstream of the *llmg_0524* ORF (−15.3 Kcals) and another one downstream of the *llmg_0526* ORF (−15.2 Kcals), suggesting a potential operonic structure for the three genes: the *llmg_0524* ORF; the *llmg_0525* ORF, which encodes a small (64 amino-acid residues) hydrophobic protein free of cysteines; and the *llmg_0526* ORF (Fig. 1a). To test our hypothesis, we performed RT-PCR assays with different primer pairs and total RNA that had been extracted from cells harvested in the exponential growth phase (Fig. 1b). The results showed that an mRNA transcript overlapped two adjacent genes (*llmg_0524-0525* and *llmg_0525-0526*). The PCR product had the same size of that obtained from genomic DNA (positive control) and was absent when RNA preparation was pretreated with a RNase (negative control). We also performed PCR with primers designed inside the *llmg_0524* and *llmg_0526* ORFs; the transcripts of this PCR product covered all three genes. No PCR amplification was obtained with primer pairs designed for the *llmg_0526* and *llmg_0527* ORFs (or for *glpT* and *llmg_0524*), a result that was consistent with the presence of stem loops (data not shown). Finally, we cloned a DNA fragment that covered the region from the middle of the *llmg_0524* ORF to the beginning of the *llmg_0526* ORF (start codon included); this fragment was inserted upstream of the promoterless *lacZ* gene of the pTCV-*lac* plasmid [33], generating plasmid P_0526_-pTCV-*lac*. After we introduced this plasmid into *L. lactis* strain MG1363, we did not detect any β-galactosidase (β-Gal) activity under the tested conditions (static, aeration, and peroxide stress growth conditions), suggesting the absence of a promoter, specifically the one upstream of the *llmg_0524* ORF. Taken together, these results indicate that the three genes are clustered in an operon and under the control of a promoter located upstream of the *llmg_0524* ORF.

**Fig. 2:**
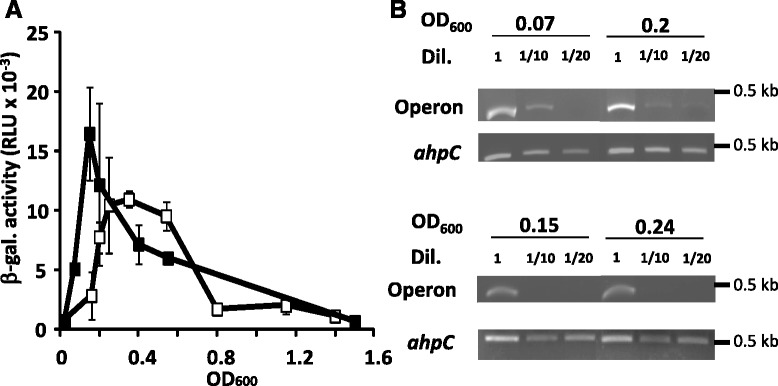
Temporal expression of the operon. **a** Expression kinetics of the operon. Cells were grown in M17glucose (black squares) or M17galactose (white squares). At regular intervals, aliquots were collected to assess β-gal activity. Values, plus standard deviations, are the means of three independent experiments. **b** Semi-quantification of the *llmg_0524* transcript. Cells were grown on glucose (top) or on galactose (bottom) and collected at certain growth phases. Total RNA was prepared for semi-quantitative RT-PCR determination. Control - *ahpC*. Dil. - dilution

To study the expression of the operon, we cloned the promoter region of *llmg_0524* ORF into the pTCV-*lac* plasmid, creating plasmid P_0524_-pTCV-*lac*, and introduced this into *L. lactis* strain MG1363. With ortho-nitrophenyl galactoside as the growth substrate and glucose as the energy source, we did not detect any β-Gal activity. However, using a more sensitive test with a fluorescent substrate (β-Glo), we managed to detect some β-Gal activity, indicating that the operon was poorly expressed. Under these conditions, we observed β-Gal induction an hour after inoculation (Fig. 2a), with a peak around OD_600_ 0.1. Beyond this point, β-Gal activity decreased progressively with further growth. When glucose was replaced by galactose as the energy source, β-Gal expression increased but appeared later (OD_600_ 0.25) in growth than was observed with glucose. Moreover, instead of having a peak, transcript quantity reached a stable plateau with minor changes until OD_600_ 0.5 and then it went down. To validate gene expression we performed semi-quantitative RT-PCR assays (Fig. 2b), which revealed relative transcript amounts that were concordant with the β-Gal activity patterns. None of the other tested growth conditions (anaerobic, aeration, and oxidative stress; data not shown) resulted in changes to the expression of the operon. In addition, deletion of *llmg_0524* or *llmg_0526* (see below) decreased two-fold expression of the operon (Additional file 2: Figure S2). We thus conclude that operon is transiently transcribed and occurs only during the early log growth phase.

### Membrane localization of Llmg_0524 and Llmg_0526

**Fig. 3:**
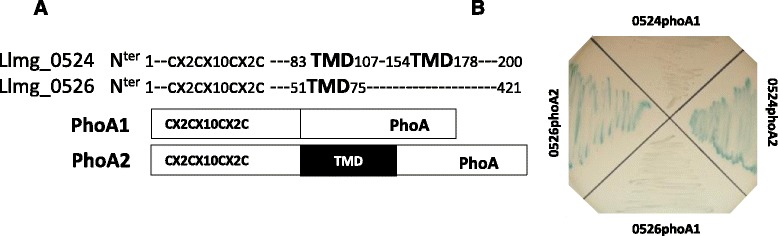
N^ter^ end of Llmg_0524 and Llmg_0526 is intracellular. **a** Schematic representation of the proteins with position of transmembrane domain (TMD) and protein fusion with PhoA. **b** Phosphatase activity of cells that overproduced the protein fusions. Fusion proteins were overproduced in a *E. coli phoA* mutant, which were then treated with arabinose. Phosphatase activity was revealed in presence of BCIP compound present in agar plates

Analyses of these proteins with topology prediction programs suggested that Llmg_0524 contained two transmembrane domains (Additional file 1: Figure S1) but the programs yielded no information about the localization of the N^ter^ domain (inside or outside). Llmg_0526 contained a transmembrane domain with the N^ter^ region, indicating that this end is probably inside the cell. To obtain direct experimental evidence regarding the membrane topology of these proteins, we performed an alkaline phosphatase (PhoA) assay [34]. PhoA is activated via the oxidation of its cysteine residue to form a disulfide bridge, a reaction which occurs mainly in the periplasm of *E. coli* cells. The PhoA assay thus consists of fusing the signal-sequence-less *phoA* gene to a target DNA sequence and then monitoring for PhoA activity; such activity should only be detectable if the target DNA addresses the resulting protein into the periplasmic space. We therefore constructed a set of plasmids that each contained a different portion of the *llmg_0524* and the *llmg_0526* ORFs fused to *phoA* (Fig. 3a), and then established the plasmids in the *E. coli phoA m*utant. Bacteria were grown on LB agar supplemented with arabinose for the production of fusion proteins and with a BCIP compound for the detection of PhoA activity. Cells that produced the PhoA2 hybrid proteins (containing the transmembrane domain) generated a strong blue color corresponding to the degradation of BCIP, while those that produced the PhoA1 constructs (lacking the transmembrane domain) displayed only weak coloration indicating that PhoA was addressed out of cytosol with *phoA2* construction (Fig. 3b). Quantification of the PhoA activity confirmed these observations, revealing the strongest activity in cells with the PhoA2 fusions (Additional file 3: Figure S3). We therefore conclude the two proteins are located in the membrane, with the N^ter^ ends located in the cytoplasm.

### Llmg_0524 and Llmg_0526 are zinc metalloproteins

As the CX_2_CX_10_CX_2_C motif in Llmg_0524 and Llmg_0526 was identical to that of the zinc metalloprotein DnaJ [9, 35], we hypothesized that our target proteins also bound a metal. To test this, we purified the N^ter^ domain of Llmg_0524 and Llmg_0526 (51 amino-acid residues) as a hybrid protein to the maltose binding protein MalE, creating MalE-0524^Nter^ and MalE-0526^Nter^, respectively. The protein MalE-lacZα corresponding to the plasmid without insert (pmal-c4x) was used as a control. After purification, the hybrid proteins were analyzed by inductively coupled plasma mass spectrometry (ICP-MS) to determine nature of metal ion if present in protein (Table 2). We found magnesium ion was present in all preparation at relative similar concentration but this ion has not been reported to bind with cysteine residues. MalE-lacZα did not contain iron or zinc metal in significant amount in contrast to other proteins. In the presence of MalE-0524^Nter^, zinc was far more abundant than iron. In preparations of MalE-0526^Nter^, although zinc was still the most abundant, there was also a quantity of iron ion. However, this protein (as well as MalE-0524^Nter^) was colorless (Additional file 4: Figure S4) and its UV-visible spectrum did not display any absorption bands in visible wavelengths, which are characteristic of iron-cysteine proteins [36]. Furthermore, when the medium was supplemented with zinc salt, the level of iron in the preparation decreased dramatically, indicating that the affinity of MalE-0526^Nter^ to iron was not specific. Altogether these results indicate that Llmg_0524 and Llmg_0526 are zinc metalloproteins.

**Table 2:** Llmg_0524 and Llmg_0526 are zinc metalloproteins

Protein	Ratio [Metal]/[protein]			Ratio [Zinc(PAR)_2_]/[protein]
	Mg	Fe	Zn	
MalE-0524^Nter^	0.21 ± 0.05	0.07 ± 0.01	0.45 ± 0.02	0.5 ± 0.07
MalE-0526 ^Nter^	0.26 ± 0.02	0.54 ± 0.05	0.79 ± 0.05	0.8 ± 0.10
MalE-lacZα	0.58 ± 0.1	0	0.04 ± 0.01	ND
+ zinc				
MalE-0524 ^Nter^	0.20 ± 0.07	0.02 ± 0.01	0.42 ± 0.08	0.4 ± 0.03
MalE-0526 ^Nter^	0.30 ± 0.11	0.03 ± 0.01	0.65 ± 0.14	0.8 ± 0.05
MalE-lacZα	0.17 ± 0.04	0	0.01 ± 0.01	ND

All Buffers were treated with Chelex. Values are the mean of three independent protein purifications. + zinc, cultures were supplemented with 0.1 mM of zinc salt. MalE-lacZα (from plasmid, pMal c4x) is used as a control, ND: not determined

### The CX_2_CX_10_CX_2_C motif binds zinc metal directly

**Fig. 4:**
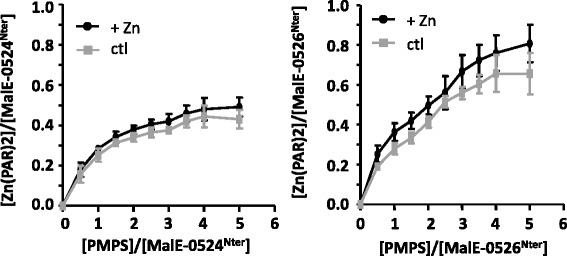
Presence of a cysteine-zinc module in proteins. The cysteine-zinc bond was characterized by PMPS-PAR assays with 20 μM of proteins. MalE-0524^Nter^ (left) or MalE-0526^Nter^ (right) were purified from cultures (ctl; grey squares) and from those that were supplemented with zinc salt (black circles) Values, plus standard deviations, are the means of three independent experiments

The ICP-MS analysis allowed us to determine the stoichiometry ratio of zinc to protein: 0.45:1 for MalE-0524^Nter^ and 0.7:1 for MalE-0526^Nter^ (Table 2). These ratios did not change when we supplemented the media with zinc salt indicating the LB broth contained enough metals to load synthesized proteins. These ratios were further confirmed by the titration of zinc ions with the chelator PAR, (4-(2-pyridylazo) resorcinol (Table 2). To demonstrate that this metal was coordinated in the proteins via the thiol group of the cysteine, we treated the proteins with p-hydroxy mercury-phenylsulfonate agent (PMPS). PMPS specifically disrupts the cysteine-zinc bond by forming a cysteine-mercury and the released zinc ion can be titrated with PAR [37]. Evidence of zinc-PAR_2_ complex formation appeared progressively with increasing amounts of PMPS in the mixture (Fig. 4). For MalE-0524^Nter^, two equivalents of PMPS versus protein were required to release around 90 % of the zinc ions, and adding more PMPS (or zinc in the medium) did not significantly change the absorbance. As there were no cysteines in the MalE portion of the fusion protein, this result meant that two cysteine residues of Llmg_0524^Nter^ were involved in binding the zinc metal. If this were the case, the protein should therefore also contain free thiols, which we determined via titration with 5,5-dithiobis(2-nitrobenzoic acid) (DTNB), a compound which reacts to free thiol groups [38]. We observed that the ratio of free cysteine:protein was close to one (Table 3). The second residue was not titrated in our assays probably because it was not accessible to DTNB due for instance to folding of Llmg_0524 fragment. In MalE-0526^Nter^, instead, four equivalents of PMPS were required to release most of the zinc from the protein, indicating a stoichiometry of 4:1 of PMPS:zinc and thus the presence of a zinc(Cys)_4_ complex. In agreement with these observations, titration with DTNB indicated a low amount of free thiol groups in our preparations (Table 3). We thus conclude that each of the two proteins harbors a zinc-cysteine module but in different coordination spheres: zinc(Cys)_2_(X)_2_ (X - unknown ligand) in Llmg_0524 and zinc(Cys)_4_ in Llmg_0526.

**Table 3:** Determination of free thiol groups in protein fusions

	MalE-524^Nter^	MalE-524^Nter^, zinc	MalE-526^Nter^	MalE-526^Nter^, zinc
Thiol (μM)	17 ± 3	17 ± 2	8 ± 1	5 ± 0.5
Thiol/protein	0.85 ± 0.15	0.85 ± 0.1	0.4 ± 0.05	0.25 ± 0.02

20 μM of protein (equivalent to 80 μM of free thiol groups) was used for thiol group determination by DTNB assays

In addition, we tested the stability of the zinc module in the presence of oxidative agents (hydrogen peroxide, cumene hydroperoxide) and a temperature of 37 °C, which affects protein folding in *L. lactis*. In the presence of ten equivalents of oxidant or 37 °C, no released zinc ions were detected by PAR (data not shown), indicating that the modules in these proteins are very stable in tested conditions.

### Llmg_0524 is required for resistance against cumene hydroperoxide and cell wall integrity

**Fig. 5:**
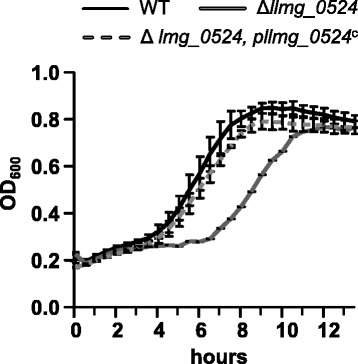
Role of *Llmg_0524* mutant in cumene hydroperoxide resistance and growth. Cells were grown in M17galactose in static conditions at 30 °C. When cell density reached OD_600_ 0.1, 0.3 mM of cumene hydroperoxide (CHP) was added to the culture. WT - black line, ∆*llmg_0524* mutant - gray line, ∆*llmg_0524* mutant carrying the plasmid encoding the full operon (p*llmg_0524* ^C^) - gray dashed line. Values, plus standard deviations, are the means of three independent experiments

**Fig. 6:**
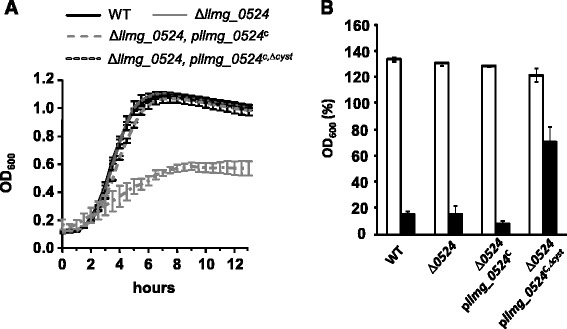
Role of Llmg_0524 in the cell wall integrity. **a** Effect on the growth of the deletion of the cysteine-rich motif in Llmg_0524. Cells were grown in M17glucose in static conditions at 30 °C. The ∆*llmg_0524* mutant carrying the plasmid p*llmg_0524* ^*c,*∆*cyst*^ is represented by a dark gray dashed line. The plasmid encoded the full operon, but lacked the region in the *llmg_0524* ORF corresponding to the cysteine motif. Values, plus standard deviations, are the means of three independent experiments (same results were obtained with galactose as an energy source). **b** Effect of lysozyme resistance of the deletion of the cysteine-rich motif in Llmg_0524. After overnight cultures, strains were washed in PBS buffer and recovered at the same OD_600_. After 60 min of incubation in absence (white bars) or in presence of lysozyme (black bars), SDS was added at 0.1 % final concentration and measured OD_600_. Values, plus standard deviation, are the means of three independent experiments. They are expressed in percent of OD_600_ measured in samples incubated 60 min in absence of lysozyme and SDS

To investigate the function of *the llmg_0524-0525-0526* operon *in vivo*, an in frame-markerless deletion of the *llmg_0524* or *llmg_0526 gene* was constructed in the chromosome of strain MG1363. As our *in silico* analyses did not yield any information about the potential functions of these proteins, we screened the mutants in several growth conditions. From these we found no clear phenotypic changes in the ∆*llmg_0526* mutant compared to its isogenic wild-type (wt) strain (data not shown). In the ∆*llmg_0524* mutant*,* instead, we found one growth condition in which the ∆*llmg_0524* mutant was more sensitive than the wt strain: growth in the presence of cumene hydroperoxide (CHP), an oxidative agent [39], and with galactose as an energy source (Fig. 5). In contrast, we did not observe any difference between the two strains treated hydrogen peroxide (data not shown). Resistance to CHP was completely restored when we transformed the ∆*llmg_0524* mutant with a plasmid expressing the wt operon (p*llmg_0524*^*C*^). Interestingly, the plasmid p*llmg_0524*^*c,*∆*cyst*^, which carried a version of the operon in which the DNA region encoding the cysteine motif of Llmg_0524 had been deleted, was unable to complement the mutant (Fig. 6). In fact, the mutant producing the truncated Llmg_0524 grew very poorly even in the absence of stress, and on glucose as energy sources as well (Fig. 6a). Then, we asked whether the modifications of lipid or cell wall synthesis were responsible of that growth defect. Addition of Tween-80, a donor of long chain fatty acids, did not have any effect on the growth. In contrast, we found that the mutant, carrying the plasmid p*llmg_0524*^*c,*∆*cyst*^, became more resistant to lysozyme, an hydrolase of peptidoglycan, than the other strains (Fig. 6b) indicating that the growth defect was due to cell wall modifications. We conclude the Llmg_0524 is linked to cell wall integrity and resistance to stressful conditions.

## Discussion

In this study we characterized Llmg_0524 and Llmg_0526, two proteins of unknown functions with the same CX_2_CX_10_CX_2_C motif. This motif bound a zinc ion. We found that the cysteine-zinc complex was stable when subjected to stress, suggesting it plays a role in protein stability rather than as a sensor. This hypothesis is supported by our observation that deletion of this motif in Llmg_0524 had serious impacts on cell growth. In addition, we did find that Llmg_0524 also increased resistance against the oxidative agent cumene hydroperoxide.

### A cysteine motif to chelate metal

In *L. lactis* strain, we demonstrated that the CX_2_CX_10_CX_2_C motif bound zinc ion. This by itself is not novel, as this motif is also found in other proteins (Additional file 5: Table S2), and in some of these, it has been experimentally demonstrated to bind this metal. For example, the DnaJ protein of *E. coli* binds zinc ion using all four cysteines of the motif, and formation of the zinc-cysteine complex is required for protein activation [9, 35]. More recently, the same motif was found in *E. coli* YciM, a membrane protein, in which the four cysteine residues were involved in metal coordination. However, YciM is proposed to bind iron ion instead of zinc [36]. From these observations alone, it was clear from this study and others (YciM, DnaJ) that the motif is used for trapping metal, but the question remained: is it possible to predict which metal, and the individual cysteine residues’ contributions to sphere coordination, based only on the motif? In this study and DnaJ, zinc ion is the major metal found suggesting that this motif may have a bigger affinity to this metal than iron. In *L. lactis*, this motif may contain zinc since in strain MG1363 level of zinc ion is 25-fold higher than that of iron whereas in *E. coli* strain zinc is present in an amount similar as iron [40, 41]. However, there are few examples describing the complex cysteine-metal to make this hypothesis true to proteins having this motif in other bacteria. With the MalE-0526^Nter^ fusion protein, PMPS-PAR assays demonstrated the presence of a zinc(Cys)_4_ module in Llmg_0526, clearly indicating that all cysteine residues were engaged in zinc coordination. Unexpectedly, however, similar tests using the fusion MalE-0524^Nter^ revealed that only two residues bound zinc. The two other cysteines of the protein were free, although we detected only one residue. That observation also indicated that MalE-0524^Nter^ lacked a disulfide bond, suggesting that the fusion protein was not redox reactive *in vitro*. However, *in vivo* these two free cysteine residues could play a role in the intact protein by forming intra- or intermolecular disulfide bridge between two Llmg_0524 or Llmg_0524 and another protein. To our knowledge, zinc usually binds four ligands, with cysteine and histidine residues as the most common ligands by far [4]. However, no histidine is present in the cytoplasmic portion of Llmg_0524, which suggests that the metal coordination of this protein is unusual. Additional experiments will be needed to determine the other ligands of zinc ion in Llmg_0524, as well as the parameters that could govern the specificity of metal.

### Role of the *llmg_0524-0525-0526 operon*

Unlike cysteine, zinc is not redox reactive in proteins. As the bound modules in Llmg_0524 and Llmg_0526 were very stable in the presence of oxidative agents or 37 °C, which affects the protein folding in *L. lactis*, it is unlikely that the module is used as a direct sensor of stress, as has been described, e.g., for the chaperone Hps33 [8]. Instead, for Llmg_0524 at least, we think that the Zn module serves to stabilize the folded structure of the protein as required for its function, perhaps for interaction with other proteins. This hypothesis is supported by the dramatic growth differences we observed between the wild-type and the truncated-Llmg_0524 mutant strain, which lacked the cysteine motif (Fig. 6). Even in the absence of stress, this strain displayed a severe growth defect. Similarly, in *E. coli*, YciM mutants lacking this motif were unstable and formed aggregates [36]. In addition, our experiments with deletion mutants suggest that this operon plays a role in cell wall integrity in particular with peptidoglycan synthesis since that mutant, producing the truncated LLmg_0524, was more resistant to lysozyme than other strains. A modification of cell wall may explain the light resistance to cumene hydroperoxide observed in the ∆*llmg_0524* mutant. Studies are in progress to characterize the modification of cell wall linked to ∆*llmg_0524* deletion. Our experiments with the ∆*llmg_0526* mutant did not produce any reproducible phenotypes even against the cumene hydroperoxide toxicity. In a protein BLAST search, this protein displays similarity to *S. aureus* TcaA, which has a cysteine motif inside, which might bind metal although this has not been confirmed [42]. Downstream of the motif, we found a transmembrane domain followed by a long extracellular chain [42]. In *S. aureus*, TcaA is induced by envelope stress and resistant to teicoplanin, an antibiotic of glycopeptide family [42]. Although they share a similar primary structure, Llmg_0526 does probably not play the same role of TcaA. In contrast to *tcaA*, *L. lactis, llmg_0524-0525-0526* operon is not induced by the envelope stress response [43].

## Conclusions

Cysteine motifs in proteins are often associated to redox activity (like thioredoxin) or iron-sulfur cluster (like [4Fe/4S]) in bacteria, but less is known about cysteine motif in chelating zinc ion. In this work, we characterized in *Lactococcus lactis*, two membrane proteins (Llmg_0524 and Llmg_0526) carrying a CX_2_CX_10_CX_2_C motif. The motif bound zinc ion although the sphere of coordination of zinc was remarkably different between the two proteins. Our works suggest that Llmg_0524, and maybe other proteins of operon, is linked to cell wall integrity. The function of this protein in the machinery of cell wall synthesis remains to uncover now, as well as its zinc module.

## Methods

### Ethics

All authors declared that no animal and human have been used in this study.

### Bacterial strains and growth conditions

The strains and plasmids used in this work are listed in Additional file 6: Table S1. For routine growth, *L. lactis* strains were grown in M17 (Oxoïd) medium that was supplemented with 0.5 % glucose, at 30 °C without shaking. The *E. coli* strain, used as a cloning host, was grown aerobically in Luria-Bertani medium (LB)*,* at 37 °C with shaking. Antibiotics were used when needed at the following concentrations: 100 μg/ml of ampicillin or 40 μg/ml of kanamycin for *E. coli* strains, 5 μg/ml of tetracycline or 0.1 μg/ml of erythromycin for *L. lactis* strains. The growth of *L. lactis* strains was evaluated as follows: i) Wild-type and mutant strains were grown in M17 media that was supplemented with sugars (galactose, glucose) from an initial cell density (OD_600_) of 0.025. When cultures reached 0.1 of OD_600_, the following compounds were added at 0.3 mM: cumene hydroperoxide or hydrogen peroxide. Growth was measured for 24 h by assessing optical densities in an Infinite M200 (Tecan) spectrophotometer.

### *In silico* analysis

*L. lactis* proteins that contained the CX_2_C motif were identified and downloaded from a genome database (Genolist server, [44]) and further analyzed with the prediction program TMHMM (Center for Biological Sequence Analysis, Technical University of Denmark, [45]) to identify membrane domains. Proteins with a probability of transmembrane domain less than 0.4 were filtered out.

### Plasmid and mutant constructions

Standard DNA recombination procedures were used, as described in Sambrook and Russell [46]. PCR amplifications were carried out using Taq Phusion DNA polymerase (Finnzymes). Amplified fragments were purified with the Purelink™ Quick PCR Purification kit or Quick Gel Extraction kit (Invitrogen). Plasmid extraction was performed with the QIAprep Spin Miniprep kit (Qiagen). Deletion mutants were constructed using the double-crossing-over method as described in Biswas et al. [47]. Using specific primers (Additional file 6: Table S1), the DNA fragments located upstream and downstream, respectively, of the target genes were amplified. These fragments were then fused in a second PCR and the DNA product was ligated into the EcoRI and XbaI sites of plasmid pBR322p + Ghost8. The insert was checked by sequencing. The modified plasmids were first established into *E. coli* strain TG1 by electroporation [48] and further into *L. lactis* strains [49]. Transformants were selected on M17 glucose that was supplemented with tetracycline at 30 °C. After integration of the plasmid into the locus at 37 °C, it was excised at 30 °C. The deletion of *llmg_0524* and *llmg_0526* in ten clones was checked by PCRs with oligos outside (upstream and downstream) of the recombination sites. For the complementation tests, the full operon, including a 500-bp open reading frame (ORF) upstream of *llmg_0524*, was amplified by PCR with primer pair 524-526-cplR and 524-526-cplF and cloned in the HindIII and SalI sites of pAK80 [50]. The resulting plasmid, p*llmg_0524*^*C*^, was then established in the ∆*llmg_0524* mutant with erythromycin as selection. A similar construct, in which only the cysteine region of *llmg_0524* was omitted, was created and designated p*llmg_0524*^*C,*∆*cyst*^.

### RT-PCR and gene expression analysis

#### RT-PCR

Total RNA was extracted from 50 ml of culture of *L. lactis* strain MG1363 at the early exponential phase (OD_600_ 0.1-0.2) using the Trizol reagent method (Invitrogen). The RNA preparation was treated with DNAse I (Fermentas) to eliminate DNA contamination (this was confirmed by the absence of PCR product following a PCR with the *ahpC* gene as the target). DNA-free RNA was translated into cDNA by the iScript™ cDNA synthesis kit (Biorad). To analyze whether *llmg_0524* and *llmg_0526* genes are transcribed in an operon, 4 μl cDNA was used for amplification by PCR with primers listed in Additional file 6: Table S1. Semi-quantitive RT-PCR. 4 μl of cDNA, pure or diluted, was used in a PCR analysis containing primers that were designed to amplify the internal region of *llmg_0524* (300 bp) and a control gene *ahpC* (419 bp) (Additional file 6: Table S1).

### Gene expression

To analyze *llmg_*0524 and *llmg_0526* gene expression, we constructed transcriptional fusions between the plasmid pTCV-*lac* [33], carrying the *lacZ* reporter gene, and the putative promoter region of each gene. The specific primers used for amplifying each promoter region (249 bp upstream of *llmg_0524* and 162 bp upstream of *llmg_0526*) are listed in Additional file 6: Table S1. After digestion with XmaI and EcoRI restriction enzymes, each PCR product was cloned into pTCV-*lac*, resulting in the creation of plasmids P_0524_-pTCV-*lac* and P_0526_-pTCV-*lac*. The ligation products were established into *E. coli* strain TG1, at which point the insertions were confirmed by PCR and sequencing using the specific oligonucleotides Vlac1 and Vlac2. The two plasmids were established into *L. lactis* strain MG1363 and transformants were selected on plates supplemented with erythromycin.

### Enzymatic assays

#### β-galactosidase fusion assays

Cells that contained plasmids P_0524_-pTCV-*lac* or P_0526_-pTCV-*lac* were grown in M17 medium supplemented with glucose or galactose. β-Galactosidase activity was quantified by luminescence in an Infinite M200 spectroluminometer (Tecan) using the β-Glo® assay system (Promega), as recommended by the manufacturer. Values were normalized to those obtained from *L. lactis* strain MG1363 carrying the plasmid pTCV-*lac*. On glucose we had time (h) after inoculation-OD_600_: 0–0.025; 1–0.075; 1.5-0.15; 2–0.2; 2.5-0.4; 3.5-0.55; 4.5-1.5; 7–2; on galactose, 1.5-0.16; 2-.2; 2.5-0.35; 4.5-.54; 7-.8; 8–1.15; 8.5-1.4. Alkaline Phosphatase (*PhoA)* assay*.* To determine the orientation of proteins, we identified the DNA regions that corresponded to the N-terminal extremity (N^ter^) of proteins with or without the transmembrane domain and cloned them into the XhoI and KpnI sites of the plasmid pHA-Zed [34]. This plasmid contained the *phoA* gene, which encodes an alkaline phosphatase enzyme under the control of an arabinose-dependent promoter region. The plasmids thus obtained were designated p524PhoA1 and p526PhoA1 (containing the N^ter^ of *llmg_*0524 or *llmg_0526*, respectively, without the membrane domain and with phoA), and p524PhoA2 and p526PhoA2 (same as PhoA1 plasmids but with the membrane domain). The plasmids were established into *E. coli* strain CC118 [34] and recombinant clones were selected with ampicillin. To measure PhoA activity in *E. coli* strains, overnight cultures were diluted in LB broth to OD_600_ 0.05 and grown until they reached OD_600_ 0.5, at which point the cultures were treated for 2 h with 0.2 % arabinose. A 100 μl aliquot was taken from each culture, washed twice, and incubated with 0.8 ml of Tris HCl buffer pH 8 (1 M) and 100 μl ρ-nitrophenyl phosphate (4 g/l) at 37 °C. When a yellow color appeared, we stopped the reactions by adding 200 μl K_2_HPO_4_ (1 M). Substrate hydrolysis was measured at OD_405_ and values are expressed in Miller units using the following formula: [OD_405_-(1.75xOD_405_)] x 1000/[incubation time (min) x OD_600_ x 0.1]. PhoA activity was visually analyzed on plates that contained LB agar supplemented with 5-bromo-4-chloro-3-indolyl phosphate (BCIP). The clones producing extracytoplasmic PhoA displayed a blue color due to the hydrolysis of BCIP in 5-bromo-4-chloro-3-indole (BCI) and inorganic phosphate under aerobic growth conditions.

### Overproduction and purification of MalE-N^ter^ fusions

For purification of the N-terminal region (N^ter^) of proteins (the first 51 amino acid residues), we used the pMal™ Protein Fusion and Purification System (Biolabs). The *llmg_0524’* fragment was amplified by PCR and cloned into the pMal-c4x vector and the product established into *E. coli* strain TG1. A transformant containing the plasmid pMal-0524^Nter^ was cultured in SOC medium supplemented or not with zinc chloride 0.1 mM. When OD_600_ was close to 0.5 we added IPTG (isopropyl 1-thiol-β-D-galactopyranoside, 0.5 mM). After 4 h of incubation, cells were collected and washed twice in 10 mM Tris–HCl pH 7.5 buffer, pretreated with Chelex resin (Sigma), and stored as a cell pellet at −80 °C. We purified MalE-0524^Nter^ by affinity chromatography according to manufacter’s procedure (Biolabs) [51] with Chelex-treated buffers. The same strategy was used to produce the fusion MalE-0526^Nter^. Protein purity was confirmed by SDS-PAGE and proteins were concentrated in spin columns (Centricon, Amicon, cut off: 10 KDa). Protein concentrations were determined with the Bradford assay method (Biorad) with bovine serum albumin as the standard.

### Zinc relative abundance determination

#### Zinc Quantification

Proteins (40 μM) were precipitated during 30 min in ice with trichloroacetic acid (TCA) at 10 %. After centrifugation (15 min, 15,000 g), the supernatant was first neutralized with NaOH and then Tris–HCl pH 7.5 was added to 0.5 M final concentration. Samples of 400 μl each were incubated for 1 h in a solution of 4-(2-pyridylazo) resorcinol (PAR) at 200 μM final concentration in a total volume of 1 ml. The formation of the Zn(PAR)_2_ complex was determined at OD_495_ (ε_495_ = 66 M^−1^. cm^−1^). PMPS-PAR assays. ρ-hydroxymercuriphenylsulfonic acid (PMPS) was used to determine if ligands formed between zinc and MalE-0524^Nter^ or MalE-0526^Nter^. Proteins were first treated with PMPS at different concentrations and a colorimetric PAR assay was used to determine the amount of zinc ion released into solution.

### Stability of cysteine/zinc complex

The stability of the complex was tested by treating proteins at 37 °C or in the presence of 10 equivalents of hydrogen peroxide or cumene hydroperoxyde at 30 °C. After 1 h incubation, the amount of zinc ion released into solution was determined by the colorimetric PAR assay.

### Free accessible thiol groups titration

Accessible thiol groups were measured using Ellman’s method [38]. Proteins (20 μM) were incubated with 60 μM of 5,5-dithiobis(2-nitrobenzoic acid) (DTNB) in 0.1 M Tris–HCl buffer (pH 8), at room temperature in the dark. After 30 min, the OD_412_ was determined. The concentration of accessible thiol groups was estimated using a standard curve of N-acetyl-L-cysteine (5–60 μM).

### ICP-MS analysis

Metal ions (Mg, Cu, Fe, Co, Mn, Zn) were identified and quantified by inductively-coupled plasma mass spectrometry (ICP-MS) (Agilent 7700x) at the Geosciences-Montpellier facility (Université Montpellier II). Cu, Co, and Mn ions were not detected in our preparations. Values were obtained in μM of metal ion and expressed in the ratio [metal ion]/[protein].

### Stress assays

Cells were cultured in M17 Glu0.5 % until saturation. Cell suspensions were diluted 100 fold in agar 0.6 % and loaded on solid M17Glu0.5 % agar plates (or M17Gal0.5 %). A disc papers soaped with compounds (30 μl of solution or 5 μl of solution) were put down on soft agar. Plates were incubated at 30 °C. Compounds tested were: NaCl 5 M, HCl 5 N, NaOH 5 N, Bacitracin 50 mg/ml, Rifampicin 50 mg/ml, DTT 2 M, H_2_O_2_ 5 M, Cumene hydroperoxide 5 M, lysozyme 0.05 up to 5 mg/ml, SDS 0.005 up to 5 %, EDTA 1 up to 25 mM, acid lactique 0.1 %, nisin 2.5 up to 25 units/ml, alone and in combination). For test at 37 °C, we incubated plates at this temperature for overnight. When we observed a difference between mutant and wild-type, tests were repeated in liquid.

### Lysozyme assays

Strains were grown in M17Glu0.5 % for overnight. Cells were washed once in PBS buffer and recovered in the buffer for OD_600_ determination. After OD_600_ adjustments, strains were treated or not with lysozyme (1 mg/ml) for 60 min at 30 °C. Then, a solution of sodium dodecyl-sulfate (SDS) was added at 0.1 % final concentration to clear the cell suspension and measured the OD_600_.

## Additional files

Additional file 1: Figure S1. Amino acid sequence of Llmg_0524 and Llmg_0526. Llmg_0524 has 200 amino-acid residues, including four cysteines in the Nter region and two transmembrane domains (TMDs). Llmg_0526 has 421 amino-acid residues, including four cysteines in the Nter region and a transmembrane domain. The cysteine residues cluster in a CX_2_CX_10_CX_2_C motif in both proteins. Cysteine amino-acid residues are in bold red; predicted membrane helices are in bold black. (PDF 304 kb) Additional file 2: Figure S2. Deletion of llmg_0524 or llmg_0526 decreases modestly operon expression. The plasmid P_0524_-pTCV-*lac* is established in mutant ∆llmg_0524 and ∆llmg_0526. Cells were grown in M17Glu0.5 up to OD_600_= 0.1 for β-galactosidase determination. Results, plus standard deviation, are means of three independent experiments. They are expressed in percentage of values of wild type strain. (PDF 171 kb) Additional file 3: Figure S3. Determination of PhoA activity of different fusion proteins. Data are the means of results, ± standard deviations, from three independent experiments. PhoA1 contains only the N^ter^ extremity whereas PhoA2 contains the N^ter^ extremity and the predicted transmembrane domain. (PDF 178 kb) Additional file 4: Figure S4. UV-visible spectra of protein fusions. 20 μM of proteins were used. Analysis was performed in 50 mM Tris–HCl buffer, pH 7.4, at room temperature with a Libra S22 spectrophotometer. (PDF 193 kb) Additional file 5: Table S2. Proteins containing the CX_2_CX_10_CX_2_C motif in various bacterial species. (PDF 512 kb) Additional file 6: Table S1. Strains and plasmids. (PDF 355 kb)

## Abbreviations

wt: Wild type
CHP: Cumene hydroperoxide
PAR: 4-(2-pyridylazo) resorcinol
N^ter^: N-terminal
DTNB: 5,5-dithiobis(2-nitrobenzoic acid)
PMPS: ρ-hydroxymercuriphenylsulfonic acid
ICP-MS: Inductively coupled plasma mass spectrometry
