# Supplementary material for: Characterization of two Lactococcus lactis zinc membrane proteins, Llmg_0524 and Llmg_0526, and role of Llmg_0524 in cell wall integrity
Source: BMC Microbiol. 2015 Oct 30;15:246. doi: 10.1186/s12866-015-0587-1 (PMC4628341; doi:10.1186/s12866-015-0587-1)
Supplement: Additional file 1: Figure S1. — Amino acid sequence of Llmg_0524 and Llmg_0526. Llmg_0524 has 200 amino-acid residues, including four cysteines in the Nter region and two transmembrane domains (TMDs). Llmg_0526 has 421 amino-acid residues, including four cysteines in the Nter region and a transmembrane domain. The cysteine residues cluster in a CX2CX10CX2C motif in both proteins. Cysteine amino-acid residues are in bold red; predicted membrane helices are in bold black. (PDF 304 kb) [file 12866_2015_587_MOESM1_ESM.pdf]

Llmg_0524
MENQPTF**C**PN**C**GKEIEAGSVF**C**TN**C**GTKMENQPANETSTANSVKPFVTEEQREILKNGATNLWE
WIVSAVKAPTKNVQENTPL**WFSWLSIILTAIFGALALGKILV**NIITNASTSVGNALGSANNSLGSLYN
QNVENTVANTANHVFGQMIFPI**IISFIILHAATILGGWLANFAIL**GDKTFTFKKMLNYYGRFMFFYLH

Llmg_0526
MENNTKF**C**PH**C**GTENKKDAAF**C**AN**C**GQSMTINQPENKEAETKEKRPVNKKM**IGIIGAVIAIFIIIGGVF**
AYINAQPKSILNAVKVNFSGYNSQGTVELLGDYQKKEIEIIGAKVGLPSSEVKKAEDSNIFSFFNSTTN
NSTKWQKFAKYFEDTRINISHSQNLSNGQKVTLKITTTLKDNPIKEETKTYTVKNLKKATTYTIESVLKD
NPVSFTGFNHFGSVKFDDDKFTVNNDNSAPTDLTNGEQIIVRLSQDYINQQKSNGKILSGTATKTLTV
ADLESSPKISNLNDLLTQEDTVVRADNESSTGDFGTTYTVTRMDSYFVGTNISSWGYSSSDDSDKGE
FSVVTIYKIVSHYNSDTDTKNDSTSYYSNGYTGLTLNNGKVDVSDLTGNNKYKGGSSSSEQAAVDQL
KSDYSSATKLN

Fig. S1.
