# Supplementary figures and images for: Characterization of two Lactococcus lactis zinc membrane proteins, Llmg_0524 and Llmg_0526, and role of Llmg_0524 in cell wall integrity
Source: BMC Microbiol. 2015 Oct 30;15:246. doi: 10.1186/s12866-015-0587-1 (PMC4628341; doi:10.1186/s12866-015-0587-1)

1

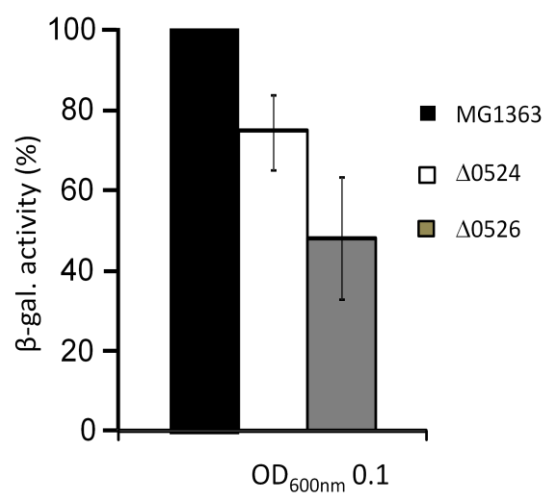

2

3

4 **Fig. S2.**

5

6

Supplement: Additional file 2: Figure S2. — Deletion of llmg_0524 or llmg_0526 decreases modestly operon expression. The plasmid P0524-pTCV-lac is established in mutant ∆llmg_0524 and ∆llmg_0526. Cells were grown in M17Glu0.5 up to OD600= 0.1 for β-galactosidase determination. Results, plus standard deviation, are means of three independent experiments. They are expressed in percentage of values of wild type strain. (PDF 171 kb) [file 12866_2015_587_MOESM2_ESM.pdf]

1

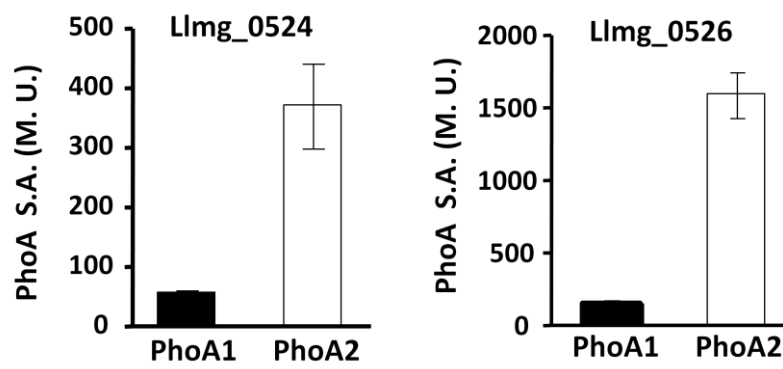

2

3 **Fig. S3.**

4

Supplement: Additional file 3: Figure S3. — Determination of PhoA activity of different fusion proteins. Data are the means of results, ± standard deviations, from three independent experiments. PhoA1 contains only the Nter extremity whereas PhoA2 contains the Nter extremity and the predicted transmembrane domain. (PDF 178 kb) [file 12866_2015_587_MOESM3_ESM.pdf]

1

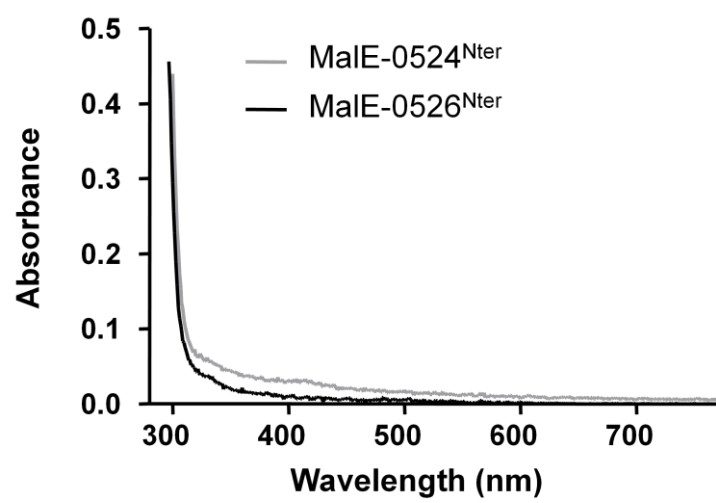

2

3

4 **Fig. S4.**

5

Supplement: Additional file 4: Figure S4. — UV-visible spectra of protein fusions. 20 μM of proteins were used. Analysis was performed in 50 mM Tris–HCl buffer, pH 7.4, at room temperature with a Libra S22 spectrophotometer. (PDF 193 kb) [file 12866_2015_587_MOESM4_ESM.pdf]
