# Supplementary material for: Characterization of two Lactococcus lactis zinc membrane proteins, Llmg_0524 and Llmg_0526, and role of Llmg_0524 in cell wall integrity
Source: BMC Microbiol. 2015 Oct 30;15:246. doi: 10.1186/s12866-015-0587-1 (PMC4628341; doi:10.1186/s12866-015-0587-1)
Supplement: Additional file 5: Table S2. — Proteins containing the CX2CX10CX2C motif in various bacterial species. (PDF 512 kb) [file 12866_2015_587_MOESM5_ESM.pdf]

Table S2. Proteins containing the $CX_2CX_{10}CX_2C$ motif in various bacterial species.

**strains**	**Membrane protein**	**Cytoplasmic protein**
L. lactis MG1363	Llmg_0524 (2 TMD), Llmg_0526 (1 TMD)	DnaJ (chaperone), RadA (DNA repair protein)
L. lactis IL1403		DnaJ, RadA
S. thermophilus LMD-9	STER_0131 (4 TMD)	DnaJ
Lb. sakei subsp *sakei* 23K	LSA1310 (5 TMD); LSA0043 (6TMD); LSA1494 (4 TMD)	DnaJ, RadA, LSA1423, LSA1707
Lb plantarum WCFS1	**Lp_2948** (1 TMD), lp_2949 (1 TMD)	DnaJ, RadA, lp_3139, lp_3215, lp_1729
B. subtilis 168	**YvbJ** (1 TMD), **YxkC** (1 TMD)	DnaJ, RadA, YczA, YqxK
E. coli K-12	FdhE (1 TMD), PqiA (8 TMD), YciM (1 TMD)	DnaJ, RadA, YejH, YhgH
Pathogens/opportunists		
E. faecalis V583	**EF_1523** (1 TMD), **EF_1542** (1 TMD)	DnaJ, RadA
L. mono. e-GDE		DnaJ, RadA
S. aureus Mu50	**TcaA**, Sav0751	DnaJ, RadA
S. agalactiae		DnaJ, RadA
S. pneumoniae R6		DnaJ
S pyogenes M1 M 1		DnaJ, RadA, M5005 Spy_0176

The transmembrane domain (TMD) is defined by TMHMM software (1); in bold, proteins that are the most similar to Llmg_0526 based on structural organization. RadA in *S. pneumoniae* and *S. thermophilus* contains only one cysteine. HMMTOP indicates no TMD in *E. coli* YciM but was demonstrated experimentally (2).

REFERENCES

1. **Krogh A, Larsson B, von Heijne G, Sonnhammer EL.** 2001. Predicting transmembrane protein topology with a hidden Markov model: application to complete genomes. J. Mol. Biol. **305:**567-580.
2. **Nicolaes V, El Hajjaji H, Davis RM, Van der Henst C, Depuydt M, Leverrier P, Aertsen A, Haufroid V, Ollagnier de Choudens S, De Bolle X, Ruiz N, Collet JF.** 2014. Insights into the function of YciM, a heat shock membrane protein required to maintain envelope integrity in *Escherichia coli*. J. Bacteriol. **196:**300-309.

Llmg_0524
MENQPTF**C**PN**C**GKEIEAGSVF**C**TN**C**GTKMENQPANETSTANSVKPFVTEEQREILKNGATNLWE
WIVSAVKAPTKNVQENTPL**WFSWLSIILTAIFGALALGKILV**NIITNASTSVGNALGSANNSLGSLYN
QNVENTVANTANHVFGQMIFPI**IISFIILHAATILGGWLANFAIL**GDKTFTFKKMLNYYGRFMFFYLH

Llmg_0526
MENNTKF**C**PH**C**GTENKKDAAF**C**AN**C**GQSMTINQPENKEAETKEKRPVNKKM**IGIIGAVIAIFIIIGGVF**
AYINAQPKSILNAVKVNFSGYNSQGTVELLGDYQKKEIEIIGAKVGLPSSEVKKAEDSNIFSFFNSTTN
NSTKWQKFAKYFEDTRINISHSQNLSNGQKVTLKITTTLKDNPIKEETKTYTVKNLKKATTYTIESVLKD
NPVSFTGFNHFGSVKFDDDKFTVNNDNSAPTDLTNGEQIIVRLSQDYINQQKSNGKILSGTATKTLTV
ADLESSPKISNLNDLLTQEDTVVRADNESSTGDFGTTYTVTRMDSYFVGTNISSWGYSSSDDSDKGE
FSVVTIYKIVSHYNSDTDTKNDSTSYYSNGYTGLTLNNGKVDVSDLTGNNKYKGGSSSSEQAAVDQL
KSDYSSATKLN

Fig. S1

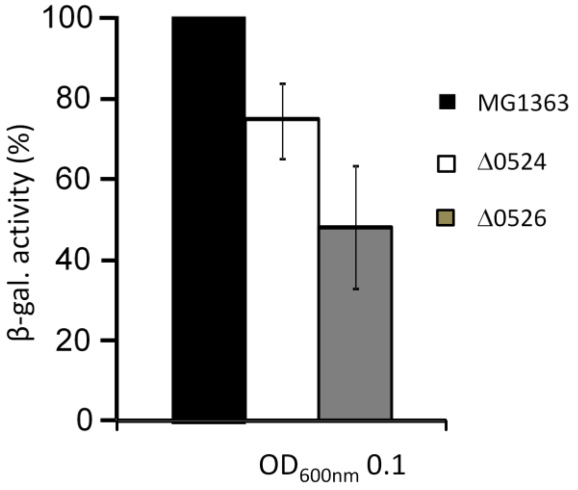

Fig. S2

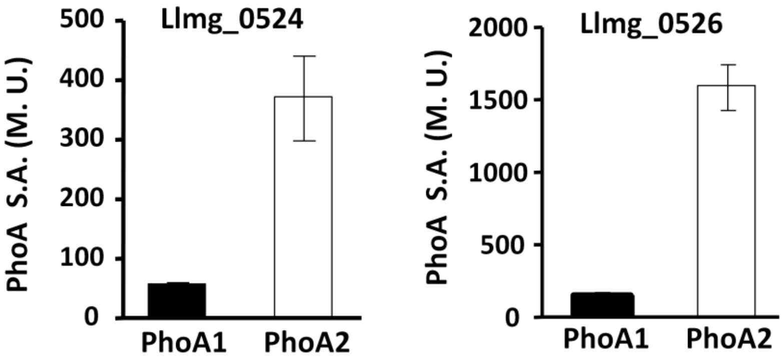
Llmg_0524
PhoA S.A. (M. U.)
500
400
300
200
100
0
PhoA1
PhoA2
Llmg_0526
PhoA S.A. (M. U.)
2000
1500
1000
500
0
PhoA1
PhoA2

Fig. S3

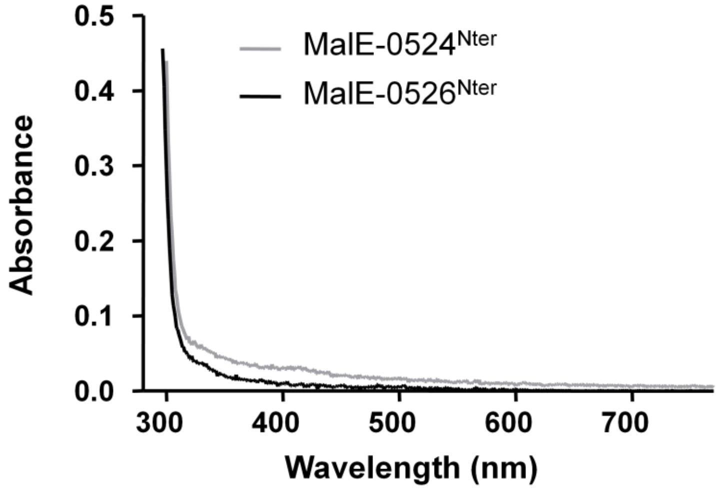
MalE-0524^{Nter}
MalE-0526^{Nter}
Absorbance
0.5
0.4
0.3
0.2
0.1
0.0
300
400
500
600
700
Wavelength (nm)

Fig. S4
