# Supplementary material for: Characterization of two Lactococcus lactis zinc membrane proteins, Llmg_0524 and Llmg_0526, and role of Llmg_0524 in cell wall integrity
Source: BMC Microbiol. 2015 Oct 30;15:246. doi: 10.1186/s12866-015-0587-1 (PMC4628341; doi:10.1186/s12866-015-0587-1)
Supplement: Additional file 6: Table S1. — Strains and plasmids. (PDF 355 kb) [file 12866_2015_587_MOESM6_ESM.pdf]

Table S1. Strains and plasmids.

Plasmid or strains	Relevant characteristic(s)	Source or reference
**Plasmids**		
pBR322pGhost8	Temperature-sensitive vector, $Am^r_{E. coli}$ $Tet^r_{L. lactis}$	(1)
pBR322pGhost8-Δ0524	pBR322pGhost8 carrying the flanking regions of *llmg_0524* gene	This study
pBr322pGhost8-Δ0526	pBR322pGhost8 carrying the flanking regions of *llmg_0526* gene	This study
pAK80	Ery^r, containing promoterless β-galactosidase gene	(2)
pAK80-*llmg_0524*^{C}	pAK80 carrying the locus of *llmg_0524-0525-0526*	This study
pAK80-*llmg_0524*$^{C,\Delta cyst}$	pAK80 carrying the locus of *llmg_0524-0525-0526* with deletion of cysteine region of Llmg_0524	This study
pHA1-YedZ	Am^r, containing an arabinose inductible promoter followed by the *phoA* gene	(3)
pHA1-524phoA1	pHA1 carrying DNA encoding the N-ter domain of *llmg_0524*	This study
pHA1-524phoA2	pHA1 carrying DNA encoding the N-ter and transmembrane domains of *llmg_0524* gene	This study
pHA1-526phoA1	pHA1 carrying DNA encoding the N-ter domain of *llmg_0526* gene	This study
pHA1-526phoA2	pHA1 carrying DNA encoding the N-ter and transmembrane domains of *llmg_0526* gene	This study
pTCV-*lac*	Shuttle vector with lacZ reporter gene, $Ery^r_{L. lactis}$ $Kan^r_{E. coli}$	(4)
P_{0524}-pTCV-*lac*	5' region of *llmg_0524* cloning into pTCV-*lac*	This study
P_{0526}-pTCV-*lac*	5' region of *llmg_0526* cloning into pTCV-*lac*	This study
pMAL-c4X	cloning vector, Am^r	New England Biolabs
pMAL-0524^{Nter}	Expression of N^{ter} MBP-tagged Llmg_0524'	This study
pMAL-0526^{Nter}	Expression of N^{ter} MBP-tagged Llmg_0526	This study
**Strains**		
E. coli		
TG1	*E. coli* TG1, *SupE hsdΔ5 thiΔ(lac-proAB) F' traD36 proAB+ lacIqlacZΔM15*, used as cloning host strain	(5)
TG1 MalE-0524^{Nter}	Am^r; TG1 carrying plasmid pMAL-0524^{Nter}	This study
TG1 MalE-0526^{Nter}	Am^r; TG1 carrying plasmid pMAL-0526^{Nter}	This study
CC118	*Δ(ara-leu)7697 ΔlacX74ΔphoA20 galE galK thi rpsE rpoB argE(am) recA1*	(3)
CC118 0524phoA1	Am^r; CC118 carrying plasmid pHA1-0524phoA1	This study
CC118 0524phoA2	Am^r; CC118 carrying plasmid pHA1-0524phoA2	This study
CC118 0526phoA1	Am^r; CC118 carrying plasmid pHA1-0526phoA1	This study
CC118 0526phoA2	Am^r; CC118 carrying plasmid pHA1-0526phoA2	This study

Lactococcus lactis		
MG1363	derived from *L. lactis* NCDO763	(6)
Δ*llmg_0524*	deletion of *llmg_0524* in MG1363	This study
Δ*llmg_0524*, pAK80-*llmg_0524*^{C}	Δ*llmg_0524* carrying pAK80-*llmg_0524*^{C}	This study
Δ*llmg_0524*, pAK80-*llmg_0524*$^{C,\Delta cyst}$	Δ*llmg_0524* carrying pAK80-*llmg_0524*$^{C,\Delta cyst}$	This study
Δ*llmg_0526*	deletion of *llmg_0526* in MG1363	This study
MG1363 P_{0524}	Ery^{r}; MG1363 carrying plasmid P_{0524}-pTCV-*lac*	This study
MG1363 P_{0526}	Ery^{r}; MG1363 carrying plasmid P_{0526}-pTCV-*lac*	This study

Primers

Use and name	Sequence (5'→3')
**Mutants**	
***llmg_0526* deletion**	
Δ0526For	GATCGGATCCATACAGTAGCAAACACTGCC
Δ0526intRev	CTGCCTGCTCTGATGACGAACTTTCTGTGCCACAATGTGGGC
Δ0526Rev	GATCTCTAGATCCCAAAACTCATTTTCGCCG
Δ0526intFor	GCCCACATTGTGGCACAGAAAGTTCGTCATCAGAGCAGGCAG
0526extFor	GGAGCGCTTGCCTTGGGAAAA
0526extRev	TGTGTAAAGGTCAATCGTCGC
***llmg_0524* deletion**	
Δ0524For	GATCGGATCCTATTGTCTTATTAATTATGGG
Δ0524intRev	CGTCCATAATAATTAAGCATCTCTTTGCCACAGTTTGGGC
Δ0524Rev	GATCTCTAGAAAAAATCGCTATTACAGCACC
Δ0524intFor	GCCCAAACTGTGGCAAAGAGATGCTTAATTATTATGGACG
0524extFor	GGCAGTTATCCTGACTTCAC
0524extRev	TGGCAGTCCAACTTTTGCACC
**Transcriptional fusion**	
llmg_0524 expression	
P_{0524}Rev	GATCCCCGGGTATTAGGATAAAATTACAATC
P_{0524}For	GATCGAATTCGTTTATGCTTTTGCTATTGC
P_{0526}For	GATCGAATTCTATCAACACCATTCATCAGC
P_{0526}Rev	GATCCCCGGGTATAATATACCGCCGGTTGC
**Llmg_0524 *orientation***	
0524phoAFor	GATCCTCGAGGATGGAAAACCAACCAACCTTTTCG
0524phoARev1	GATCGGTACCGAGAGGGGAGTATTTTCTTGTACG
0524phoARev2	GATCGGTACCGACGAACCAAGTGCATTCCCGAC
**Llmg_0526 *orientation***	

0526phoAFor	GATCCTCGAGGATGGAAAACAACACAAAATTTTGC
0526phoARev1	GATCGGTACCGACATTTTTTTATTTACTGGACG
0526phoARev2	GATCGGTACCGATGTTGAGTTATTTGTCGTTGAGTTG
**Complementation**	
***llmg_0524* and *llmg_0526* Complementation**	
524-526-cplF	GATCAAGCTTGTTTATGCTTTTGCGGGTATTGC
524-526-cplR	GATCGTCGACGTCTTTTGAACTTTTTCGGG
524ΔC intRev	CTGATTCTCCATTTTTGTTCCAAAGGTTGGTTGGTTTTCCAT
524ΔC intFor	ATGGAAAACCAACCAACCTTTGGAACAAAAATGGAGAATCAG
**N^{ter}0526 purification**	
malE-Nter0526For	GATCGAATTCATGGAAAACAACACAAAATTTTGCC
malE-Nter0526Rev	GATCGTCGACTTATTACATTTTTTTATTTACTGGAC
**N^{ter}0524 purification**	
malE-Nter0524For	GATCGAATTCATGGAAAACCAACCAACCTTTTGCC
malE-Nter0524Rev	GATCGTCGACTTATTATTGTTCTTCAGTAACAAAAGG
**Operon**	
524-opFor	GGGTTGTTCAATCTTATGGCT
524-opFor2	CAACCAACCTTTTGCCCAAAC
524-opRev	TTTTCCCAAGGCAAGCGCTCC
524-525opFor	GGCGATAAAACTTTTACATTC
524-525opRev	TAAAAAATGAAATAAATCCCC
525-526opFor	GCATGGCTTGCTAATAGTGCG
525-526opRev	TTTCTGTGCCACAATGTGGGC
526-opFor	GGGGATTTTGGAACAACTTAT
526-opRev	AATCAATAAATCATTTCTCCC

REFERENCES

1. **Biswas I, Gruss A, Ehrlich SD, Maguin E.** 1993. High-efficiency gene inactivation and replacement system for gram-positive bacteria. J. Bacteriol. **175:**3628-3635.
2. **Israelsen H, Madsen SM, Vrang A, Hansen EB, Johansen E.** 1995. Cloning and partial characterization of regulated promoters from *Lactococcus lactis* Tn917-lacZ integrants with the new promoter probe vector, pAK80. Appl. Environ. Microbiol. **61:**2540-2547.

3. **Drew D, Sjostrand D, Nilsson J, Urbig T, Chin CN, de Gier JW, von Heijne G.** 2002. Rapid topology mapping of *Escherichia coli* inner-membrane proteins by prediction and PhoA/GFP fusion analysis. Proc. Natl. Acad. Sci. U. S. A. **99:**2690-2695.

4. **Poyart C, Trieu-Cuot P.** 1997. A broad-host-range mobilizable shuttle vector for the construction of transcriptional fusions to beta-galactosidase in gram-positive bacteria. FEMS Microbiol. Lett. **156:**193-198.

5. **Sambrook J, Russell DW.** 2001. Molecular cloning: a laboratory manual, 3rd ed. Cold Spring Harbor Laboratory Press, Cold Spring Harbor, N. Y.

6. **Gasson MJ.** 1983. Plasmid complements of *Streptococcus lactis* NCDO 712 and other lactic streptococci after protoplast-induced curing. J. Bacteriol. **154:**1-9.
